# Effect of *DNMT3A* R882H Hot Spot Mutations on *DDX43* Promoter Methylation in Acute Myeloid Leukemia

**DOI:** 10.1155/2024/9625043

**Published:** 2024-05-21

**Authors:** Tahere Tabatabaei, Mohammad Reza Rezvany, Bahare Ghasemi, Farzane Vafaei, Masoumeh Kiani Zadeh, Farhad Zaker, Arash Salmaninejad

**Affiliations:** ^1^Department of Hematology and Blood Transfusion, School of Allied Medical Sciences, Iran University of Medical Sciences, Tehran, Iran; ^2^Blood Transfusion Research Center, High Institute for Research and Education in Transfusion Medicine, Isfahan, Iran; ^3^Blood Transfusion Research Center, High Institute for Research and Education in Transfusion Medicine, Tehran, Iran; ^4^Regenerative Medicine, Organ Procurement and Transplantation Multi-Disciplinary Center, Razi Hospital, School of Medicine, Guilan University of Medical Sciences, Rasht, Iran; ^5^Department of Medical Genetics, Faculty of Medicine, Tehran University of Medical Sciences, Tehran, Iran

## Abstract

Epigenetic alterations have been observed in many hematological malignancies, including acute myeloid leukemia (AML). Many of these alterations result from mutations in DNA methyl transferase (DNMT) enzymes, disabling them to methylate target genes in a proper way. In this case-control study, we investigated the association between R882H mutation in *DNMT3A* gene and *DDX43* gene methylation in patients with AML. 47 AML patients and 6 controls were included in this study. After DNA extraction, amplification refractory mutation system (ARMS)-PCR was used to evaluate R882H mutations in *DNMT3A* gene. The high-resolution melting (HRM) method was used to determine the methylation changes of the *DDX43* gene promoter. R882H mutation was only found in 10.6% (5 out of 47) of AML patients. The frequency of *DDX43* gene methylation was significantly higher in patients without R882H mutations compared to patients with R882H mutations (*P* < 0.05). The DNMT3A R882H mutation is typically present in a minority of AML patients. Nevertheless, this mutation is associated with a reduced frequency of methylation in the DDX43 promoter region.

## 1. Introduction

Acute myeloid leukemia (AML) is an aggressive malignancy of monocyte or granulocyte cells contributing to about 80% of all blood cancers in adults, and it is a type of leukemia characterized by the uncontrolled growth of abnormal precursor cells and halted differentiation. Until 2017, it was the most prevalent leukemia subtype in adults in the US but was later surpassed by CLL (chronic lymphocytic leukemia). In 2019, an estimated 21,450 adults were expected to be diagnosed with AML, with males comprising 11,650 cases and females comprising 9,800 cases. AML is responsible for the largest proportion (62%) of leukemia-related deaths [1, 2]. The incidence of this malignancy is 4.3 per 100,000 annually in the United States (US). The five-year survival rate of people with AML is dependent on several factors, varying from less than 30% (for people ≥ 20) to 70% (for people older than 20). Given the aggressive entity of this malignancy and its considerable incidence, it is crucial to understand the mechanisms underlying this cancer [1].

The mechanisms contributing to the development of AML include a wide spectrum of genetic mutations and epigenetic aberrations. Mutations in epigenetic regulators such as DNA methyl transferase (DNMT) enzymes and those involving in histone modifications can occur very early before malignancy manifestation and facilitate clonal expansion of hematopoietic stem cells [3]. Generally, DNMT enzymes direct DNA methylation transferring methyl group from S-adenosyl methionine (SAM) to cytosine on 5′-carbon positions. DNA methylation causes chromatin stability and represses gene expression whereas DNA demethylation causes chromatin instability and activation of gene transcription. Dysregulation of these epigenetic mechanisms can activate oncogenes and/or inactivate tumor suppressor genes, thereby promoting cancerous phenotype [4, 5]. The members of the DNMT family include DNMT1, DNMT2, DNMT3A, DNMT3B, DNMT3L, and DNMT3C, which all cause nucleic acid methylation at different times of the cell cycle. DNMT1 concerns with maintenance methylation, ensuring the transmission of lineage-specific DNA methylation patterns during DNA replication [6, 7]. DNMT2 contributes to methylation of transfer RNA (tRNA) instead of DNA [8]. DNMT3A and DNMT3B play an important role in *de novo* methylation during the embryonic development. In fact, DNMT3B set genomic methylation pattern in early embryonic stages, whereas DNMT3A is expressed in the later developmental stages as well as postnatal [5, 9]. DNMT3L has an inactive catalytic domain and can only cooperate with DNMT3A in DNMT3L-DNMT3A heterodimers. Also, it is required for imprinting and spermatogenesis [10, 11]. The exact mechanism of DNMT3C in human is not yet elucidated; however, its *de novo* methylation activity has been shown to be necessary in protecting murine germ cells from deleterious activity of transposons [12].

Given the crucial role of DNMTs in the regulation of gene expression, mutations in them can result in inappropriate activation/inactivation of cancer-related genes, causing malignant phenotype. Early studies suggest that *DNMT3A* mutations are involved in hematological malignancies such as myelodysplastic syndrome (MDS), adult early T-cell precursor acute lymphoblastic leukemia (ETP-ALL), and AML [13–17]. In AML patients, more than half of the *DNMT3A* mutations are heterozygous missense mutations within the catalytic domain at arginine 882 (R882) [18]. Mutations in this hot spot have a dominant-negative effect and are associated with higher levels of peripheral blood hemoglobin and poor prognosis [19]. In fact, these mutations keep DNMT3A from methylation of several target genes, resulting in hypomethylation and activation of them. One such gene is DEAD (Asp-Glu-Ala-Asp) box polypeptide 43 (DDX43, also known as *HAGE*), a member of the cancer/testis antigen (CTAs) family [20]. DDX43 was first identified together with sarcoma antigen (*SAGE*) as a tumor-specific CTA gene in a human sarcoma cell line [21]. DDX43 has been identified as being overexpressed in a range of solid tumors including those affecting the salivary gland, colon, brain, lung, and prostate, as well as in hematologic malignancies like chronic myeloid leukemia and multiple myeloma [22–29]. Recently, abnormal hypomethylation of *DDX43* promoter has frequently been reported in hematologic malignancies, including CML, MDS, and AML [30]. Hypomethylation of *DDX43* gene promoter results in activation of its transcription, which leads to induction of RAS protein expression and signaling. In addition, studies suggest that the RNA helicase activity of this gene has a key role in the resistance of ABCB5+ malignant melanoma stem cells to IFNalpha treatment by promoting SOCS1 expression [31]. Given the high frequency of *DDX43* hypomethylation in hematologic malignancies due to *DNMT3A* mutations [22], nevertheless, the pattern of *DDX43* methylation has not been well studied in AML; in this study, we aim to evaluate the relation between *DNMT3A* R882H mutations and *DDX43* methylation status in Iranian patients with AML.

## 2. Method and Material

### 2.1. Patients and Controls

In this study, 47 newly diagnosed AML patients with ≥30% blast were enrolled, who referred to Shariati Hospital from June 2020 to December 2021. The patients' AML type was classified according to French-American-British (FAB) criteria. Karyotyping was carried out for all the patients. We also included a control group, consisting of 6 healthy individuals. Written informed consent was obtained from all the participants. The local research ethics committee of Iran University of Medical Sciences (ethical code IR.IUMS.REC1395.9411264004) approved this study. The clinical and laboratory characteristics of the participants are summarized in Table 1.

Peripheral blood specimens were taken from all the participants and collected in EDTA-containing tubes. For peripheral blood mononuclear cell (PBMC) separation, the blood samples were diluted 1 : 2 times in phosphate buffered saline (PBS) and then subjected to density gradient centrifugation (1.08 g/mol) by Ficoll-Hypaque (Pharmacia, Freiburg, Germany) in falcon 15 mL conical centrifuge tubes at 700 g for 20 min. PBMC samples were then transferred to fresh 1.5 mL microcentrifuge tubes and washed with PBS at 500 g for 10 minutes.

### 2.2. DNA Extraction

DNA was extracted from each PBMC sample using QIAamp Blood DNA Mini Kit (QIAGEN, Hilden, Germany) according to the manufacturer's instructions. The quality of extracted DNA was evaluated by electrophoresis on 1% agarose using loading dyes.

### 2.3. Bisulfite Conversion

DNA samples were subjected to modification by sodium bisulfite for methyl-sensitive high-resolution melting curve (MS-HRM). After obtaining DNA concentration by NanoDrop ND-1000 (Nanodrop Technologies, Wilmington, Delaware, USA), we used 1 mg/mL of each DNA sample for bisulfite treatment using EZ DNA Methylation-Golden kit (Zymo Research Crop. Irvine, CA, USA). The final elution volume was 20 *μ*L.

### 2.4. ARMS-PCR

For detection of R882H mutation in exon 23 of *DNMT3A* gene, we performed tetra-primer amplification refractory mutation system (ARMS)-PCR. For this purpose, *DNMT3A* DNA sequence was taken from the NCBI (http://www.ncbi.nlm.nih.gov) website. Primers were designed with Primer1 online software (http://primer1.soton.ac.uk/primer1.html). Primers' details are given in Table 2. The reaction volume was 20 *μ*L, consisting of 10 *μ*L hot start 2X master mix blue, 0.5 *μ*L outer forward primer, 1.5 *μ*L outer reverse primer, 1.5 *μ*L inner forward, 0.4 *μ*L inner reverse, and 1.5 *μ*L genomic DNA. Primer was prepared in 10 nm/*μ*L consistency. Tetra ARMS-PCR was performed by a BIO-RAD T100TM thermal cycler. The thermal cycling condition was as follows: 95°C initial heating for 5 minutes, 32 cycles of 95°C denaturation temperature for 1 minute, 61.5°C annealing temperature for 45 seconds and 72°C extension temperature for 45 seconds, and finally, a step of 72°C for 5 minutes.

### 2.5. Sanger Sequencing

To confirm our results regarding the presence or absence of R882H mutation detected by ARMS-PCR, we sent 5 mutated and 3 unmutated samples to Pishgam Biotech Co. for Sanger sequencing and confirming ARMS-PCR method results.

### 2.6. MS-HRM Assay

Alteration in methylation of *DDX43* promoter was evaluated by semiquantitate high-resolution melting (HRM) in Light Cycler 96 instrument (Roche Diagnostics GmbH, Mannheim, Germany). Primers for promoter sequence were as follows: forward 5′-GTTTCGTGCGGGTTTTTTAAGTAG-3′ and reverse 5′-CGTCCAACTCTACACCACCTA-3′. The promoter sequence was obtained from https://genome.ucsc.edu and then evaluated in Metprimer online software (https://www.urogene.org/methprimer/).

Each reaction consisted of 3 *μ*L 5X Hot FIREPOL Eva Green qPCR Mix, 0.5 *μ*L forward primer, 0.5 *μ*L reverse primer, and 1 *μ*L bisulfite-modified DNA. The final volume got to 15 *μ*L with distilled water. The protocol was set on 95°C preincubation for 15 minutes, 45 cycles of 95°C for 15 seconds, 60°C annealing temperature for 20 seconds, and 72°C extension temperature for 20 seconds, and then, 10 cycles were added to MS-HRM consisting of 95°C heating for 1 minute, 40°C for 1 minute, 65°C for 1 minute, and continuous heating to 95°C at a ramp rate of 0.05°C per second. Fully methylated and unmethylated standards were purchased from the Pasteur Institute of Iran (Tehran, Iran). For creating a standard curve, we prepared different dilutions (0, 25, 50, 75, and 100%) and used them in our run work. Sample curves were then compared with standard curves.

### 2.7. Statistical Analysis

Data are presented as median and mean ± SD. Data normality was assessed using the Shapiro–Wilk test. Student's *t*-test and Mann–Whitney *U* tests were used to compare quantitative data. Fisher's exact test and Chi-squared test were carried out to compare frequencies. *P* < 0.05 was considered as statistically significant. Statistical analysis was performed using STATA (v.14) software.

## 3. Results

### 3.1. Patients' Characteristics

In this study, 38% (18/47) of the patients were women and 61% (29/47) were men. The average age of patients at the time of diagnosis was 47.91 ± 18.45, whereas the average age of the controls was 41.2. The median age of the patients and the controls was 39 and 40, respectively. AML subtypes were classified based on FAB criteria, and M4 (16/47) and M2 (10/47) were the commonest types, respectively. WBC was higher while RBC and platelets were significantly lower than healthy donors (*P* < 0.05) as shown in Table 1.

### 3.2. ARMS-PCR

**Figure 1:**
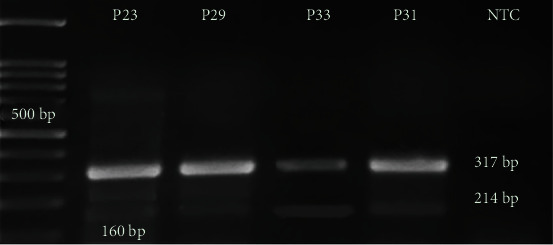
Tetra ARMS-PCR. Patients 23 and 29 are heterozygous in R882H of DNMT3A gene (2 out of 5 patients have been shown in the picture, P23 and P29). They showed 3 bounds in 160, 214, and 317 bp. Patients 33 and 31 are wild-type homozygous without mutation in R882H. They have only 2 bounds in 160 and 317 bp (2 out of 42 have been shown here, P31 and P33). 100 bp ladders were used in 1.5% agarose gel. P: patient; Neg: negative.

According to the results from our tetra ARMS-PCR, five patients were heterozygote for R882H mutation, where A was substituted by G in nucleotide 2853 located in exon 23. This status was indicated by observing three bands on gel electrophoresis. Arginine to histidine change results in a negative domain on wild-type protein. The control band (317 bp) was observed in all samples. In samples with mutation, 2 other bands (214 bp and 160 bp) were observed in addition to the control band. Other 42 patients had only 2 bonds in 317 and 160 bp (Figure 1).

### 3.3. Sanger Sequencing

**Figure 2:**
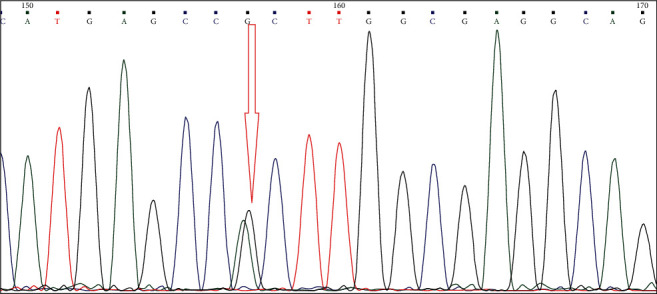
Sanger sequencing results: heterozygote mutation (*G* > *A*) shows two peaks at the 2853 nucleotide site.

As stated in materials and methods, we performed Sanger sequencing on five mutated and three unmutated samples to confirm the results from the ARMS-PCR method. Our sequencing results confirmed the genotyping results of ARMS-PCR and demonstrated that 5 patients showed *A* > *G* transition at nucleotide 2853, which results in R882H missense mutation. This mutation is located in the catalytic domain and results in a negative domain and the lack of activity (Figure 2).

We also evaluated the associations between R882H mutations and clinical and laboratory characteristics of the patients, including WBC, PLT, RBC, and AML subtypes. Three of *DNMT3A* R882H-mutated patients were in M4 subtype, one in M2 subtype, and one in M1 subtype. Therefore, M4 and M2 are the commonest subtypes in R882H-mutated patients, as were in unmutated patients. Three patients with the mutation were male, and 2 were female. All of them were normal in karyotypes, and only one had unfavorable risk del (5)(q22; q34) with higher median WBC and PLT. Also, there was not any association with R882H mutation and other clinical and laboratory characteristics.

### 3.4. HRM Assay

**Figure 3:**
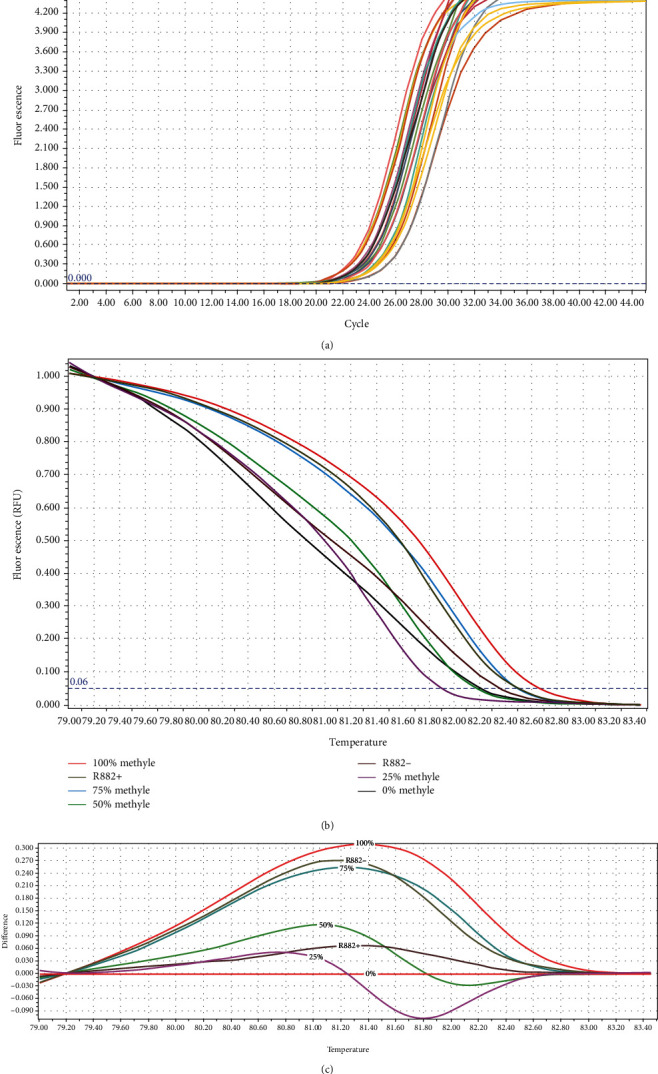
(a) Amplification curves of patients' samples. (b) Normalized melting curve of DDX43 serial standard dilution including 0, 25, 50, 75, and 100. Sample with R882H mutation (R882H+) showed methylation between 25 and 50% methylation. Sample without R882H mutation (R882H-) showed methylation between 75 and 100% methylation. (c) Differential graph of each standard sample against the unmethylated sample. Methylation range between R882H+ sample and R882H- sample was compared to the other standard sample.

HRM assay can distinguish a 0.01°C difference in the melting temperature of DNA molecules. Serial dilutions of fully methylated and unmethylated standards were prepared, and each of the 25, 50, 75, and 100% graphs was compared with the unmethylated graph. The melting curves are given in Figure 3.

### 3.5. The Relation between *DNMT3A* R882H Mutation and *DDX43* Promoter Methylation

Subsequent to genotyping and evaluation of the methylation status, we compared these two states to see whether there is any association between R882H mutation in *DNMT3A* gene and promoter methylation in *DDX43* gene.

The data regarding the distribution of patients (with or without R882H mutation) and controls in various methylation status groups are given in Table 3. As shown in this table, the major number of patients without R882H mutation (30 out of 42) showed 75-100% methylation in *DDX43* promoter, whereas only 1 out of 5 patients with R882H mutation showed this methylation status. Therefore, the distribution of mutated and unmutated patients in the methylation status groups was significantly different (*P* < 0.05). In addition, none of the healthy controls revealed 75-100% methylation, demonstrating a statistically significant difference between patients and controls (*P* < 0.05).

## 4. Discussion

In this study, using ARMS-PCR and Sanger sequencing, we evaluated the frequency of R882H mutations in exon 23 of *DNMT3A* gene in newly diagnosed AML patients and healthy controls. In addition, we assessed the association between R882H mutations and methylation status in *DDX43* gene promoter. Our results demonstrated that 10.6% of the patients showed R882H mutation, and this mutation was significantly associated with decreased methylation in *DDX43* gene (*P* < 0.05), suggesting the possible role of *DNMT3A* function in promoter methylation and transcriptional inactivation of *DDX43*. Our result may shed a light on the mechanism of action of *DNMT3A* mutation R882H, which is a prognostic factor for AML patients.

DNMTs play an important role in genomic methylation during the cell cycle. DNMT-mediated methylation of gene promoters results in transcription repression and reduced gene expression. Aberrant methylation of various genes has been demonstrated in solid tumors and various hematological malignancies such as MDS, CML, and AML [32].

DNMT3A is an important member of DNMTs and plays an important role in *de novo* methylation of DNA [5, 9]. Mutations in *DNMT3A* gene have been reported frequently in AML, and R882H mutations are the most frequent ones [33]. R882H mutations disappear after complete remission in AML patients [34]; therefore, this factor can be used for therapeutic monitoring and even the type of treatment choice.

Ley et al. found R882H mutations in 37 of 281 (13.1%) AML patients with massively parallel sequencing of *DNMT3A* gene, of which 27 resulted in arginine to histidine at this position R882H [15]. In another study on 63 Egyptian AML patients, 17 patients (27%) showed mutations, of which 11 patients (61.1%) were burdened with R882H mutation [35]. On the other spectrum, in a Chinese study, Yamashita et al. reported that, from 870 adult patients receiving standard induction therapy, 74 patients (8.51%) showed R882H mutations, investigated by pyrosequencing [36]. Also, Lin et al. showed that DNMT3A R882H mutations were found more frequently among monoblastic leukemia compared to nonmonoblastic leukemia (*P* = 0.041). Their results further confirmed the specificity of DNMT3A R882H mutations in monocytic lineage; furthermore, 86.6% of R882H mutations were observed in patients with normal karyotypes [34]. The discrepancy in the prevalence of R882H mutations in AML patients may be due to differences in patients' numbers, differences in race and ethnicity, the method used to detect the mutations, and other genetic and environmental factors. In the present study, we found that 10.6% of the patients showed R882H mutations in a heterozygous state, which is almost consistent with the previous studies, all demonstrating that <20% of the patients harbor R882H mutations.

In the present study, we also evaluated the associations between R882H mutation and clinical and laboratory characteristics of our AML patients. In line with previous studies [37], we did not find any associations between R882H and patient characteristics, including, age, gender, and WBC, PLT, and RBC counts. However, Ghannam et al. reported the association between higher age and WBC count and R882H mutations in 63 cytogenetically normal AML patients [35].

DDX43 is a new member of the RNA helicase family that contributes to pre-mRNA splicing, RNA metabolism, and RNA degeneration [38]. Other family members such as DDX51, DDX5, and DDX53 play important roles in several cancers [39, 40]. In 2014, Lin et al. reported that the *DDX43* gene promoter gets hypomethylated frequently in AML and the DDX43 protein level is significantly higher in hypomethylated patients than in methylated patients. Studies have also shown that this event is a favorable prognostic factor in AML [37] as well as MDS [9, 30]. On the other hand, Roman-Gomez et al. found a poor prognosis in CML patients [26]. Given the frequent studies reporting the hypomethylation of *DDX43* promoter in hematological malignancies, and the significance of R882H mutations in DNMT3A, we hypothesized that there may be some relations between these two events. We detect a significant association between R882H and low methylation status. Given the favorable prognostics of *DDX43* hypomethylation which has been reported by the previous studies [9, 30, 37] and the association of this hypomethylation with R882H mutation in *DNMT3A* in our study, it may be concluded that R882H mutation may be a favorable prognostic factor for AML patients. Previous studies have reported that the prognostic impact of DNMT3A R882H versus non-R882H mutations in AML is inconclusive [18, 41, 42]. A recent study showed that *DNMT3A* R882H mutations confer unique clinical characteristics in MDS, including a high risk of AML transformation [43]. Similar to *DNMT3A* R882H mutation, *DDX43* has been shown to be involved in tumor cell development in many types of cancers [40]. However, DDX43-related transformation may not be so devastating as Lin et al. reported that *DDX43* hypomethylation was associated with favorable/intermediate-risk groupings in AML [37]. Also, DDX43 expression increases following exposure to demethylating agent 5-aza-deoxycytidine which can help following response to therapy [37].

In conclusion, considering all of the above-mentioned studies, as well as our study, it may be concluded that *DNMT3A* R882H mutations, which are usually observed in <20% of AML patients, may have a role in transforming blood cells to AML, but this event is a favorable prognostic factor. The reason for this conclusion may be that this mutation may result in decreased methylation of *DDX43* promoter. In summary, the *DDX43* gene is activated by promoter hypomethylation and *DDX43* hypomethylation may be a favorable prognostic factor in AML. Further studies, however, are needed to confirm the association between *DNMT3A* R882H mutation and *DDX43* promoter hypomethylation and to elucidate the exact mechanism by which DDX43 functions in AML.

## Figures and Tables

**Table 1:** Clinical and laboratory characteristics of AML patients and controls.

Laboratory characteristic	Patients	Controls	*P* value
Median age (min–max)	39 (18-95)	40 (24-68)	0.97
Gender (male/female)	29/18	4/2	
Median WBC (∗10 mL)	63	7.5	0.005
Median hemoglobin (gr/L)	8	13.5	0.0005
Plt (∗10)	30	274.5	0.0003
FAB classification		—	0.4
M0	6	—	
M1	7	—	
M2	10	—	
M3	2	—	
M4	16	—	
M5	6	—	
Patient karyotype^∗^			
NK-AML	20	NA	
Favorable	3	NA	
Unfavorable	8	NA	
Intermediate	1	NA	
Unclassified	3	NA	
Missed (unknown)	12	NA	

^∗^Cytogenetic classification was based on WHO criteria. NK-AM: normal karyotype-AML. The meaning of a favorable response generally indicates an overall survival rate of about 50-60% for patients who achieve remission after therapy for AML with abnormalities involving chromosome 16,t(8;21), and perhaps even for the group that has deletion involving the long arm of chromosome 9. AML patients with unfavorable-risk cytogenetic abnormalities account for 16-30% of younger adult patients and have poor response to standard treatment, with only 32-55% achieving a complete response. The intermediate-risk cytogenetic subclass of AML includes cytogenetically normal (CN) and AML with other cytogenetic abnormalities and accounts for approximately 60% of all AML patients. NA: not available.

**Table 2:** Primers for tetra ARMS-PCR.

Primer name	Primer sequence	Melting temperature (°C)
Forward inner	5′-CCACTATACTGACGTCTCCAACATGAGACG-3′	69
Reverse inner	5′-GCCCAGCAGTCTCTGCCTCGCCACGT-3′	78
Forward outer	5′-GAGTTGGTGGGTGTGAGTGCCCCTGT-3′	73
Reverse outer	5′-CTTTGTGTCGCTACCTCAGTTTGCCCCC-3′	73

**Table 3:** The distribution of *DNMT3A* R882H mutated/unmutated patients and healthy controls in *DDX43* promoter methylation groups.

Samples	DDX43 promoter methylation (%)				*P* value^∗^
	0-25	25-50	50-75	75-100	
Patients					
Without R882H mutation	2	5	5	30	
With R882H mutation	1	3	—	1	0.020^a^
Total	3	8	5	30	
Controls	—	3	3	—	0.035^b^

^∗^Chi-squared. ^a^Comparison between patients with R882H mutations and those without this mutation. ^b^Comparison between total number of patients and controls.

## Data Availability

The data that support the findings of this study are available on request from the corresponding author.

## References

[1] Shallis R. M., Wang R., Davidoff A., Ma X., Zeidan A. M. (2019). Epidemiology of acute myeloid leukemia: recent progress and enduring challenges. *Blood Reviews*.

[2] Juliusson G., Antunovic P., Derolf Å. (2009). Age and acute myeloid leukemia: real world data on decision to treat and outcomes from the Swedish Acute Leukemia Registry. *Blood*.

[3] Chaudry S. F., Chevassut T. J. T. (2017). Epigenetic guardian: a review of the DNA methyltransferase DNMT3A in acute myeloid leukaemia and clonal haematopoiesis. *BioMed Research International*.

[4] Jones P. A., Baylin S. B. (2007). The epigenomics of cancer. *Cell*.

[5] Smith Z. D., Meissner A. (2013). DNA methylation: roles in mammalian development. *Nature Reviews Genetics*.

[6] Bröske A.-M., Vockentanz L., Kharazi S. (2009). DNA methylation protects hematopoietic stem cell multipotency from myeloerythroid restriction. *Nature Genetics*.

[7] Zhang W., Xu J. (2017). DNA methyltransferases and their roles in tumorigenesis. *Biomarker Research*.

[8] Goll M. G., Kirpekar F., Maggert K. A. (2006). Methylation of tRNA^Asp^ by the DNA methyltransferase homolog Dnmt2. *Science*.

[9] Chen T., Ueda Y., Dodge J. E., Wang Z., Li E. (2003). Establishment and maintenance of genomic methylation patterns in mouse embryonic stem cells by Dnmt3a and Dnmt3b. *Molecular and Cellular Biology*.

[10] Bourc'his D., Xu G. L., Lin C. S., Bollman B., Bestor T. H. (2001). Dnmt3L and the establishment of maternal genomic imprints. *Science*.

[11] Kaneda M., Okano M., Hata K. (2004). Essential role for *de novo* DNA methyltransferase Dnmt3a in paternal and maternal imprinting. *Nature*.

[12] Barau J., Teissandier A., Zamudio N. (2016). The DNA methyltransferase DNMT3C protects male germ cells from transposon activity. *Science*.

[13] Walter M. J., Ding L., Shen D. (2011). Recurrent *DNMT3A* mutations in patients with myelodysplastic syndromes. *Leukemia*.

[14] Neumann M., Heesch S., Schlee C. (2013). Whole-exome sequencing in adult ETP-ALL reveals a high rate of *DNMT3A* mutations. *Blood*.

[15] Ley T. J., Ding L., Walter M. J. (2010). *DNMT3A* mutations in acute myeloid leukemia. *New England Journal of Medicine*.

[16] Yan X.-J., Xu J., Gu Z.-H. (2011). Exome sequencing identifies somatic mutations of DNA methyltransferase gene *DNMT3A* in acute monocytic leukemia. *Nature Genetics*.

[17] Figueroa M. E., Lugthart S., Li Y. (2010). DNA methylation signatures identify biologically distinct subtypes in acute myeloid leukemia. *Cancer Cell*.

[18] Marcucci G., Metzeler K. H., Schwind S. (2012). Age-related prognostic impact of different types of *DNMT3A* mutations in adults with primary cytogenetically normal acute myeloid leukemia. *Journal of Clinical Oncology*.

[19] Yang L., Shen K.’. F., Zhang M.’. L. (2019). Clinical features and microRNA expression patterns between AML patients with DNMT3A R882 and frameshift mutations. *Frontiers in Oncology*.

[20] Salmaninejad A., Zamani M. R., Pourvahedi M., Golchehre Z., Hosseini Bereshneh A., Rezaei N. (2016). Cancer/testis antigens: expression, regulation, tumor invasion, and use in immunotherapy of cancers. *Immunological Investigations*.

[21] Martelange V., De Smet C., De Plaen E., Lurquin C., Boon T. (2000). Identification on a human sarcoma of two new genes with tumor-specific expression. *Cancer Research*.

[22] Adams S., Sahota S., Mijovic A. (2002). Frequent expression of *HAGE* in presentation chronic myeloid leukaemias. *Leukemia*.

[23] Nagel H., Laskawi R., Eiffert H., Schlott T. (2003). Analysis of the tumour suppressor genes, FHIT and WT-1, and the tumour rejection genes, BAGE, GAGE-1/2, HAGE, MAGE-1, and MAGE-3, in benign and malignant neoplasms of the salivary glands. *Molecular Pathology*.

[24] Mathieu M. G., Linley A. J., Reeder S. P. (2010). HAGE, a cancer/testis antigen expressed at the protein level in a variety of cancers. *Cancer Immunity*.

[25] Condomines M., Hose D., Raynaud P. (2007). Cancer/testis genes in multiple myeloma: expression patterns and prognosis value determined by microarray analysis. *The Journal of Immunology*.

[26] Roman-Gomez J., Jimenez-Velasco A., Agirre X. (2007). Epigenetic regulation of human cancer/testis antigen gene, HAGE, in chronic myeloid leukemia. *Haematologica*.

[27] Liggins A. P., Lim S. H., Soilleux E. J., Pulford K., Banham A. H. (2010). A panel of cancer-testis genes exhibiting broad-spectrum expression in haematological malignancies. *Cancer Immunity*.

[28] Amer N. N., Khairat R., Hammad A. M., Kamel M. M. (2023). DDX43 mRNA expression and protein levels in relation to clinicopathological profile of breast cancer. *PLoS One*.

[29] Tan H., Wang W., Zhou C. (2023). Single-cell RNA-seq uncovers dynamic processes orchestrated by RNA-binding protein DDX43 in chromatin remodeling during spermiogenesis. *Nature Communications*.

[30] Chen Q., Lin J., Yao D. M. (2012). Aberrant hypomethylation of *DDX43* promoter in myelodysplastic syndrome. *British Journal of Haematology*.

[31] Linley A. J., Mathieu M. G., Miles A. K., Rees R. C., McArdle S. E. B., Regad T. (2012). The helicase HAGE expressed by malignant melanoma-initiating cells is required for tumor cell proliferation *in vivo*. *The Journal of Biological Chemistry*.

[32] Thorgeirsson S. S., Grisham J. W. (2002). Molecular pathogenesis of human hepatocellular carcinoma. *Nature Genetics*.

[33] Zare-Abdollahi D., Safari S., Movafagh A. (2015). A mutational and expressional analysis of *DNMT3A* in acute myeloid leukemia cytogenetic subgroups. *Hematology*.

[34] Lin J., Yao D. M., Qian J. (2011). Recurrent *DNMT3A* R882 mutations in Chinese patients with acute myeloid leukemia and myelodysplastic syndrome. *PLoS One*.

[35] El Ghannam D., Taalab M. M., Ghazy H. F., Eneen A. F. (2014). *DNMT3A* R882 mutations in patients with cytogenetically normal acute myeloid leukemia and myelodysplastic syndrome. *Blood Cells, Molecules, and Diseases*.

[36] Yamashita Y., Yuan J., Suetake I. (2010). Array-based genomic resequencing of human leukemia. *Oncogene*.

[37] Lin J., Chen Q., Yang J. (2014). *DDX43* promoter is frequently hypomethylated and may predict a favorable outcome in acute myeloid leukemia. *Leukemia Research*.

[38] Cordin O., Banroques J., Tanner N. K., Linder P. (2006). The DEAD-box protein family of RNA helicases. *Gene*.

[39] Camats M., Guil S., Kokolo M., Bach-Elias M. (2008). P68 RNA helicase (DDX5) alters activity of *cis*- and *trans*-acting factors of the alternative splicing of H-Ras. *PLoS One*.

[40] Fuller-Pace F. V., Moore H. C. (2011). RNA helicases p68 and p72: multifunctional proteins with important implications for cancer development. *Future Oncology*.

[41] Gaidzik V. I., Schlenk R. F., Paschka P. (2013). Clinical impact of *DNMT3A* mutations in younger adult patients with acute myeloid leukemia: results of the AML Study Group (AMLSG). *Blood*.

[42] Renneville A., Boissel N., Nibourel O. (2012). Prognostic significance of DNA methyltransferase 3A mutations in cytogenetically normal acute myeloid leukemia: a study by the Acute Leukemia French Association. *Leukemia*.

[43] Jawad M., Afkhami M., Ding Y. (2022). DNMT3A R882 mutations confer unique clinicopathologic features in MDS including a high risk of AML transformation. *Frontiers in Oncology*.

